# Dose-volume parameters predict radiation pneumonitis after induction chemoradiotherapy followed by surgery for non-small cell lung cancer: a retrospective analysis

**DOI:** 10.1186/s12885-019-6359-9

**Published:** 2019-11-26

**Authors:** Kuniaki Katsui, Takeshi Ogata, Kenta Watanabe, Norihisa Katayama, Junichi Soh, Masahiro Kuroda, Katsuyuki Kiura, Yoshinobu Maeda, Shinichi Toyooka, Susumu Kanazawa

**Affiliations:** 1 Department of Proton Beam Therapy, Okayama University Graduate School of Medicine, Dentistry and Pharmaceutical Science, 2-5-1 Shikata-cho, Kita-ku, Okayama, 700-8558 Japan; 2 Department of Radiology, Iwakuni Clinical Center, Iwakuni, Yamaguchi, 740-8510 Japan; 3 Department of Radiology, Okayama University Hospital, 2-5-1 Shikata-cho, Kita-ku, Okayama, 700-8558 Japan; 4 Division of Thoracic Surgery, Department of Surgery, Kindai University Faculty of Medicine, 377-2 Ohno-Higashi, Osakasayama, Osaka, 589-8511 Japan; 5 Department of Radiological Technology, Graduate School of Health Sciences, Okayama University, 2-5-1 Shikata-cho, Kita-ku, Okayama, 700-8558 Japan; 6 Department of Allergy and Respiratory Medicine, Okayama University Hospital, 2-5-1 Shikata-cho, Kita-ku, Okayama, 700-8558 Japan; 7 Hematology, Oncology and Respiratory Medicine, 2-5-1 Shikata-cho, Kita-ku, Okayama, 700-8558 Japan; 8 General Thoracic Surgery and Breast and Endocrinological Surgery, 2-5-1 Shikata-cho, Kita-ku, Okayama, 700-8558 Japan; 9 Radiology, Okayama University Graduate School of Medicine, Dentistry and Pharmaceutical Science, 2-5-1 Shikata-cho, Kita-ku, Okayama, 700-8558 Japan

**Keywords:** Radiation pneumonitis, Mean lung dose, Lower lobe, Induction chemoradiotherapy, Non-small cell lung cancer

## Abstract

**Background:**

The relationship between lung dose-volume histogram (DVH) parameters and radiation pneumonitis (RP) associated with induction concurrent chemoradiotherapy (CCRT) followed by surgery in patients with non-small cell lung cancer (NSCLC) is unclear, particularly when concerning irradiation of the whole lung prior to resection. We performed this study to identify factors associated with grade ≥ 2 RP in such patients.

**Methods:**

Patients who received induction CCRT (chemotherapy: cisplatin and docetaxel; radiotherapy: 46 Gy/23 fractions) between May 2003 and May 2017 were reviewed. The mean lung dose (MLD) and the percentage of the lung volume that received ≥5 Gy (V5) and ≥ 20 Gy (V20) were calculated. Factors associated with the development of grade ≥ 2 RP were analyzed.

**Results:**

One hundred and eight patients were included in this study, 34 (31.5%) of whom experienced grade ≥ 2 RP. A V20 ≥ 21%, an MLD ≥10 Gy, and a lower lobe tumor location were significant predictors of grade ≥ 2 RP on univariate analysis (*p* = 0.007, 0.002, and 0.004, respectively). Moreover, an MLD ≥10 Gy and lower lobe location were significant predictors of grade ≥ 2 RP on multivariate analysis (*p* = 0.026 and 0.0043, respectively). The cumulative incidence rates of grade ≥ 2 RP at 6 months were 15.7 and 45.6% in patients with MLDs < 10 Gy and ≥ 10 Gy, respectively, and were 23.5 and 55.6% in patients with upper/middle lobe- vs. lower lobe-located tumors, respectively.

**Conclusions:**

MLD and lower lobe location were predictors of grade ≥ 2 RP in patients who received induction CCRT. It is necessary to reduce the MLD to the greatest extent possible to prevent the occurrence of this adverse event.

## Background

Definitive concurrent chemoradiotherapy (CCRT) is considered a standard therapy for patients with locally advanced stage III non-small-cell lung cancer (NSCLC); the concurrent administration of docetaxel and cisplatin has shown promising results [1]. However, long-term local control rates remain inadequate, leading to various treatment strategies that may include surgical resection [2]. Meta-analyses of individual participant data regarding preoperative chemotherapy have shown improved survival for patients with stage IB–IIIA NSCLC [3]. Moreover, some researchers have attempted to incorporate radiotherapy into induction therapy. At our institution, Toyooka et al. demonstrated that the 3- and 5-year overall survival rates of patients who received induction CCRT were significantly higher than those of patients who received induction chemotherapy [4]. The phase III INT 0139 randomized controlled trial that compared induction CCRT plus subsequent lobectomy to definitive CCRT without surgery in patients with stage III NSCLC found that the overall survival was poorer in the latter group than in the former [5].

Radiation pneumonitis (RP) is a notable adverse event after thoracic radiotherapy. A recent breakthrough phase III trial of durvalumab after CCRT showed significantly longer progression-free survival in patients administered durvalumab than in those administered a placebo [6]. However, one of the exclusion criteria in that trial was grade 2 or higher RP resulting from a previous CCRT regimen.

While previous studies have investigated the correlation between the dose-volume histogram (DVH) parameters and RP in patients who received definitive CCRT [7–12], there are (to our knowledge) only 2 published studies investigating the relationship between RP and DVH parameters in patients who underwent induction CCRT. One study by Takahashi et al. that investigated the relationship between RP and DVH parameters revealed that the irradiation of ≥12% of the remnant (post-resection) lung volume with at least 20 Gy (V20) and lobectomy were significant factors of RP on univariate analysis [13]. Moreover, our own previous study was the first to show that the V20 to the remnant lung was a predictive factor for RP on multivariate analysis [14]. Analyses of DVH-CCRT associations usually investigate the V20 and mean lung dose (MLD), although the preoperative resection target volume may change after induction CCRT. However, the aforementioned 2 studies did not show that the V20 and MLD to the total lung (i.e., pre-resection) were significantly associated with grade ≥ 2 RP.

In the present study, we investigated the relationship between whole-lung DVH parameters and the development of grade ≥ 2 RP in patients receiving induction CCRT for NSCLC.

## Methods

### Patients

Data from patients with histologically confirmed NSCLC who received induction CCRT between May 2003 and May 2017 at our institution were reviewed retrospectively. Induction CCRT mainly administered to patients with resectable bulky N2–3 tumors, although some patients also underwent this procedure based on the surgeon’s discretion. The eligibility criteria for this study were as follows: radiotherapy administered at a dose of 46 Gy in 23 fractions, chemotherapy with cisplatin/docetaxel administered concurrently with radiotherapy, and the completion of the preplanned surgery after induction CCRT. All procedures followed were in accordance with the ethical standards laid down in the 1964 Declaration of Helsinki and its later amendments. Written informed consent was obtained from each patient before treatment. Moreover, through notifications displayed in the outpatient ward and on the institution’s website, patients were provided the option to opt-out of this study. The institutional review board of Okayama University Graduate School of Medicine, Dentistry and Pharmaceutical Sciences and Okayama University Hospital approved this study (approval number: 1809–018).

### Treatment

Physical examination, chest radiography, chest and abdominal computed tomography (CT), brain magnetic resonance imaging, bone scans, and fluorodeoxyglucose positron emission tomography-CT were performed to obtain detailed data for staging, which was determined using the 7th edition of the TNM classification of malignant tumors. Treatment planning for radiotherapy was performed using 2–10 mm-thick CT scans obtained at 2–10 mm intervals while patients were in the supine position without respiratory arrest and tumor tracking. The gross tumor volume (GTV) included the primary tumors and clinically diagnosed metastatic nodal stations. The clinical target volume included the GTV with a 5–10 mm margin plus the non-metastatic subcarinal and ipsilateral hilar nodal stations; prophylactic nodal irradiation to the non-metastatic nodal stations was not performed after 2015. The planning target volume included the clinical target volume with a 5–10 mm margin and consideration for the internal and setup margins. The internal margin was determined at the discretion of the attending doctor based on X-ray fluoroscopy images. All patients underwent 3-dimensional treatment planning using the Xio computer software version 4.8.0 (Elekta, Sweden) with a superposition dose calculation algorithm for heterogeneity correction. The prescribed dose at the isocenter was 46 Gy, with 2 Gy per fraction once daily, using a 10 MV photon beam delivered by a linear accelerator (Mevatron Primus or ONCORCanon, Japan).

The chemotherapy regimen was cisplatin/docetaxel in all patients, and was concurrently administered with radiotherapy based on the OLCSG 0007 trial [1]. The preplanned surgery was performed approximately 1 month after the completion of radiotherapy.

### Evaluation

The DVH parameters of the total lung minus the gross tumor were considered. Lung contouring was performed automatically using the CT-based threshold; the trachea and bronchi were manually excluded. The RP grade was determined according to the Common Toxicity Criteria for Adverse Events (CTCAE) version 4.0. The diagnostic criteria for radiation pneumonitis were as follows: Grade 1: Asymptomatic; clinical or diagnostic observations only; intervention not indicated; Grade 2: Symptomatic; medical intervention indicated; limiting instrumental activities of daily living (ADL); Grade 3: Severe symptoms; limiting self-care ADL; oxygen indicated; Grade 4: Life-threatening respiratory compromise; urgent intervention indicated; and Grade 5: Death. The MLD and the percentage of the lung volume that received more than 5 Gy (V5) and 20 Gy (V20) were analyzed as DVH parameters; the relationships between these parameters and the incidences of grades ≥2 RP were investigated via univariate analysis with Fisher’s exact test as well as multivariate analysis using the Cox proportional hazards model. Before performing Fisher’s exact test, we used the median cutoff value to convert continuous parameters to binomial ones.

Statistical significance was defined as *p* < 0.05; factors found to be significant on univariate analysis were subjected to multivariate analysis. The cumulative incidence rate of RP was determined using the Kaplan-Meier method stratified by factors found to be significant on multivariate analysis. The R software, version 3.2.0 (R Foundation for Statistical Computing) was used for all statistical analyses.

## Results

One hundred and eight patients were included in this study; their characteristics are shown in Table 1. One stage IV patient had axillary lymph node metastasis that was surgically removed. All the patients received induction CCRT with a dose of 46 Gy. Ten patients who did not undergo surgery after induction CCRT due to reasons, such as appearance of distant metastasis before surgery in seven patients, deterioration of general condition in one patient, deterioration of general condition due to RP in one patient, and refusal of surgery after remission of RP in one patient, were excluded in this study. The median interval to surgery was 5.8 weeks (range: 3.1–13.0 weeks) after completion of radiotherapy. Lobectomy, bilobectomy, and pneumonectomy were performed in 86, 14, and 8 patients, respectively. The median follow-up period after completion of radiotherapy was 42.9 months (range: 2.0–152.1). The median V20 and MLD were 20.7% (range: 7.0–38.5%) and 10.3 Gy (range: 3.5–14.5 Gy), respectively.

**Table 1:** Patient characteristics

		%
Age (years)		
Median (range)	62 (34–79)	–
Sex		
Male	80	74
Female	28	26
ECOG-PS		
0	64	59
1	42	39
2	1	1
Smoking History (Brinkmann Index)^a^		
Median (range)	760 (0–3120)	–
Lobe		
Upper	72	67
Middle	9	8
Lower	27	25
Laterality		
Right	62	57
Left	46	43
Histology		
Adenocarcinoma	50	46
Squamous cell carcinoma	42	39
Undifferentiated carcinoma	1	1
Non-small cell carcinoma	15	14
C-stage		
IIA	4	4
IIB	9	8
IIIA	66	61
IIIB	28	26
IV	1	1
Operation		
Lobectomy	86	80
Bilobectomy	14	13
Pneumonectomy	8	7
FEV1 (l)^a^		
Median (range)	2.52 (1.40–4.06)	–
Tumor size (mm)		
Median (range)	44.33 (15.64–107.00)	–
GTV volume (cc)		
Median (range)	66.25 (10.17–601.44)	–
Number of lymph nodes		
Median (range)	2 (0–11)	–
Resected lung volume (cc)		
Median (range)	734.54 (52.82–1759.25)	–
Residual lung volume (cc)		
Median (range)	2503.98 (953.54–4415.03)	–
V5 (%)		
Median (range)	32.0 (10.8–54.9)	–
V20 (%)		
Median (range)	20.7 (7.0–38.5)	–
MLD (Gy)		
Median (range)	10.3 (3.5–17.7)	–
Period from completion of RT to surgery (weeks)		
Median (range)	5.9 (3.1–13.0)	–

*ECOG-PS* Eastern Cooperative Oncology Group performance status, *FEV1* Forced expiratory volume in 1 s, *GTV* Gross tumor volume, *RT* Radiotherapy.

^a^These factors have missing values

**Fig. 1:**
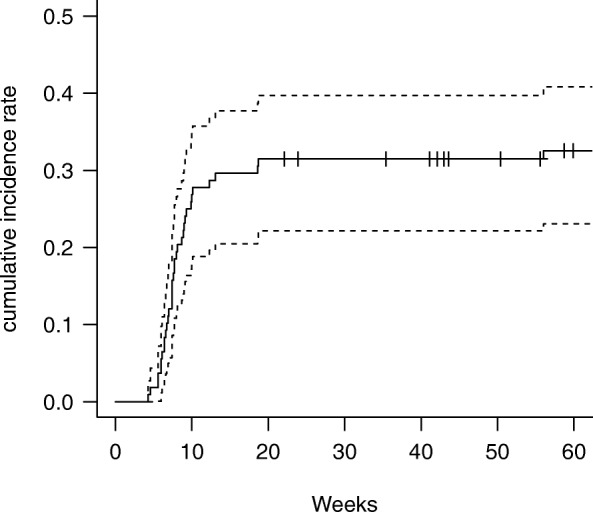
Cumulative incidence rate of grade ≥ 2 radiation pneumonitis after completion of radiotherapy. The broken lines indicate 95% confidence intervals

A total of 30, 43, 32, and 3 patients experienced RP with grades 0, 1, 2 and 3, respectively. The median period from completion of induction CCRT to the onset of grade ≥ 2 RP was 7.6 weeks (range: 4.3–56 weeks). Of the 35 patients with grade ≥ 2 RP, 6 experienced this toxicity before surgery. All 6 patients developed G2 RP. The mean values of V5, V20, and MLD were 31.6, 21.3%, and 10.5 Gy in the group that developed ≥2 RP before surgery and 37.7, 24.8%, and 12.1 Gy in the group that developed ≥2 RP after surgery, respectively. There was no significant difference between the two groups (*p* = 0.269, 0.386, and 0.404, respectively). Figure 1 shows the cumulative incidence rate of grade ≥ 2 RP (32.4, 95% confidence interval: 23.7–42.1) at 6 months.

The results of univariate and multivariate analyses of factors associated with grade ≥ 2 RP are shown in Table 2. A V20 ≥ 21%, an MLD ≥10 Gy, and a tumor location in the lower lobe were found to be statistically significant factors on univariate analyses (*p* = 0.007, 0.002, and 0.004, respectively). There was a tendency for RP to occur more easily in the group with reduced residual lung volume than in the group with normal residual lung volume (*p* = 0.064). Patient age, sex, performance status, smoking status, resection range, forced expiratory volume in 1 s, and V5 were not significantly associated with the development of grade ≥ 2 RP. On multivariate analysis, the MLD and tumor location were found to be significantly associated with grade ≥ 2 RP (*p* = 0.026 and 0.004, respectively). According to the cut-off value established by Tsujino et al. [8], the cumulative incidence rates at 6 months were 23.7 and 50.0% in patients with V20 s ≤25 and > 25%, respectively.

**Table 2:** Univariate and multivariate analyses of factors associated with grade ≥ 2 radiation pneumonitis

Factor		N	Univariate *p*-value	Odds ratio (95% CI)	Multivariate *p*-value
Age (years)	< 62	18/51	0.681	–	NE
	≥62	17/57			
Sex	Male	24/80	0.482	–	NE
	Female	11/28			
ECOG-PS ^a^	0	24/64	0.215	–	NE
	1–2	11/43			
Smoking history (Brinkmann Index)^a^	< 760	17/49	0.673	–	NE
	≥760	16/55			
Lobe	Lower lobe	15/27	0.004	3.75	0.004
	Upper/Middle lobe	20/81		(1.39–10.5)	
Laterality	Right	21/62	0.836	–	
	Left	14/46			
Surgery	Lobectomy	26/86	0.350	–	NE
	Bilobectomy	7/14			
	Pneumonectomy	2/8			
FEV1 (L)^a^	< 2.5	13/48	0.293	–	NE
	≥2.5	20/53			
Tumor size (mm)	< 44	22/54	0.099	–	NE
	≥44	13/54			
GTV volume (cc)	< 66	20/53	0.305	–	NE
	≥66	15/55			
Number of lymph	< 2	10/44	0.095	–	NE
Nodes	≥2	25/64			
Resected lung	< 730	16/53	0.684	–	NE
volume (cc)	≥730	19/55			
Residual lung	< 2500	22/53	0.064	–	NE
volume (cc)	≥2500	13/55			
V5 (%)	< 32	13/55	0.064	–	NE
	≥32	22/53			
V20 (%)	< 21	11/55	0.007	3.27	0.349
	≥21	24/53		(1.31–8.62)	
MLD (Gy)	< 10	9/51	0.002	3.86	0.026
	≥10	26/57		(1.50–10.8)	

*CI* Confidence interval, *NE* Not entered, *ECOG-PS* Eastern Cooperative Oncology Group Performance Status, *FEV1* Forced expiratory volume in 1 s, *GTV* Gross tumor volume, *MLD* Mean lung dose of the lung, *V20* Percentage of the lung volume receiving at least 20 Gy.

^a^These variables have missing values

**Fig. 2:**
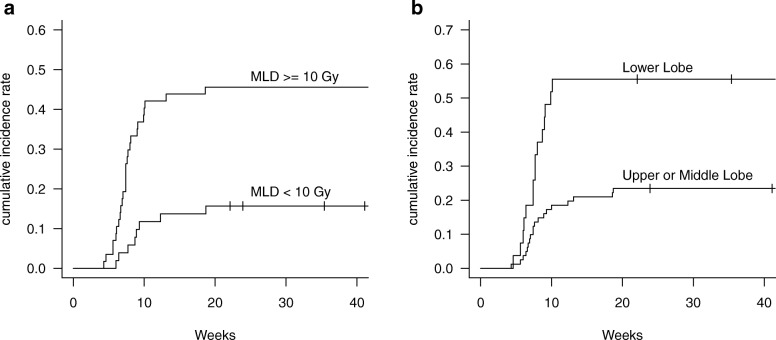
Subgroup analysis of the incidence of grade ≥ 2 radiation pneumonitis after completion of radiotherapy. **a** Cumulative incidence rate grade ≥ 2 radiation pneumonitis stratified according to the mean lung dose (MLD). **b** Cumulative incidence rate of grade ≥ 2 radiation pneumonitis stratified according to the tumor location in the lung

Figure 2 shows the cumulative incidence rates of grades ≥2 RP stratified according to MLD and tumor location. The rates of grade ≥ 2 RP at 6 months were 15.7 and 45.6% in patients with MLDs < 10 Gy and ≥ 10 Gy, respectively, and were 23.5 and 55.6% in patients with upper/middle lobe location and lower lobe location, respectively. In groups with ≥2 RP and < 2 RP, the five-year disease-free survival rates were 55.1 and 62.9%, respectively, and the five-year overall survival rates were 81.3 and 73.5%, respectively. There were no significant differences between the two groups (*p* = 0.6 and 0.5, respectively).

## Discussion

RP often occurs in patients undergoing definitive radiotherapy for NSCLC, although grade 5 RP is rare [8, 9, 12]. Among the DVH parameters, V20 and MLD are the most common predictors of RP in patients undergoing definitive radiotherapy with or without chemotherapy. Graham et al. reported that V20 was the sole independent predictor of RP [7], as none of their patients developed this adverse effect when V20 was less than 22, 8% developed RP grade ≥ 2 RP when V20 was 22–31, and 13% did when V20 was 32–40%. One-half of their patients were treated with radiotherapy alone. While the incidence rate of RP has risen in the era of CCRT, Tsujino et al. found that the V20 was the sole factor associated with grade ≥ 2 RP after definitive CCRT [8]; the 6-month cumulative incidences of grade ≥ 2 RP were 8.7, 18.3, 51, and 85% in patients with V20 s of < 20%, 21–25%, 26–30, and > 31%, respectively. The 6-month cumulative incidence of grade ≥ 2 RP among their patients was 14% in those with V20 s up to 25 and 63% in those with V20 s ≥26%. Data from a large meta-analysis of predictors of RP showed that the rates of symptomatic RP were 18.6 and 30.3% in patients with V20 s < 20% and 20–29.99%, respectively, and the rates of fatal pneumonitis were 2.9 and 3.5% in patients with V20 s of 30–40% and ≥ 40%, respectively [9]. In our study, the cumulative rates of grade ≥ 2 RP at 6 months were 23.7 and 50.0% in patients with V20 s of ≤25 and > 25%, respectively, according to the cut-off value established by Tsujino et al. [8]. While comparing the incidences of RP between previous studies and ours should be performed with caution, owing to the reliance on different CTCAE versions and V20 cut-off values, the grade ≥ 2 RP rates in our study appeared to be within range of those previously reported in patients who received definitive CCRT. On the other hand, none of our patients experienced fatal PR. In our study, however, we targeted patients who were likely to tolerate surgery before starting induction CCRT; because definitive CCRT is usually indicated for unresectable locally advanced NSCLC, our patients may have been in relatively better condition than those investigated in previous studies.

MLD is also an important predictor of RP in patients undergoing definitive CCRT. Barriger et al. reported that the rates of grade ≥ 2 RP were 2.2 and 19% in patients with MLDs < 18 Gy and > 18 Gy, respectively [12]. Palma et al. also found the MLD to be a predictor of RP in the patients ≤65 years treated with carboplatin/paclitaxel chemotherapy using recursive partitioning analysis [9]; the rates of RP were 0–9% and 41–48% among patients with MLDs < 10 Gy and ≥ 10 Gy, respectively. In our study, the RP rates at 6 months were 15.7 and 45.6% in patients with MLDs < 10 Gy and ≥ 10 Gy, respectively, which are consistent with Palma et al.*’s* data. Dang et al. found that the MLD is a predictor of grade ≥ 2 RP using multivariate logistic regression analysis, with an odds ratio of 1.42 (95% confidence interval: 1.28–1.58) [11]. Hence, the fact that MLD and V20 were predictors of RP in our study is consistent with other published data of patients who underwent definitive CCRT.

Takahashi et al. investigated the relationship between RP and DVH parameters among patients with NSCLC treated with induction CCRT and surgery, and found that a V20 to the resected lung (i.e., post-lobectomy) of ≥12% was a significant predictor of grade ≥ 2 RP [13]. However, neither the MLD nor V20 to the whole lung was significantly associated with grade ≥ 2 RP in their study, even on univariate analysis. However, V20 and MLD to the whole lung were statistically significant factors in our univariate analyses, whereas MLD was also significant on multivariate analysis. To our knowledge, our study is the first to show that MLD and V20 to the whole lung are predictors of grade ≥ 2 RP in patients undergoing induction CCRT.

Previous investigators have reported that a lower lobe tumor location is predictive of grade ≥ 2 RP development in patients receiving definitive chemoradiotherapy (CRT) [9–11]. For example, Park et al. found that, among patients treated with 3-dimensional conformal radiotherapy as part of definitive CRT for NSCLC, 40% of those with lower lobe tumor locations developed grade ≥ 2 RP compared to 25% of those with upper and middle lobe locations; the difference was significant on univariate analysis [10]. Dang et al. also revealed that a lower lobe tumor location was significantly associated with grade ≥ 2 RP on univariate analysis [11]. Their group investigated 369 consecutive patients with stage III NSCLC who were treated with CRT, and found that the incidences of RP in 235/134 patients with upper/lower lobe tumor locations were 164/76 for grades 0–1, 48/32 for grade 2, and 23/26 for grade 3. The rates of grade ≥ 2 RP in patients with upper and lower lobe tumor locations were 30.2 and 43.3%, respectively. While our data are comparable to those of previous studies, the rates of grade ≤ 2 RP were 23.5 and 55.6% in patients with upper/middle lobe and lower lobe tumor locations, respectively; hence, the difference in grade ≥ 2 RP incidence between patients with upper and lower lobe tumor locations was greater than that in previous studies. We also found that tumor location was a significant predictor of grade ≥ 2 RP on multivariate analysis. In Palma et al.*’s* study, fatal RP (which occurred in 1.9% of patients) was found to be associated with tumor location, with 1, 0, and 5% of RP-related fatalities occurring in patients with tumors in the upper, middle, and lower lobes, respectively (*p* = 0.007) [9]. Further studies are required to understand why a lower lobe tumor location is an independent predictor of RP in patients undergoing induction CCRT. There was a tendency for ≥2 RP to occur more easily in the group with reduced residual lung volume than in the group with normal residual lung volume. This result is consistent because a patient with small lung volume is likely to develop symptoms of respiratory disease. In the study by Takahashi et al., there was no significant difference in the residual lung volume [11]. However, the small sample size was one of the limitations of that study, and the difference in results between our study and that of Takahashi et al. may be due to the difference in sample size.

Various methods have been tried to improve radiotherapy dose distribution. Intensity-modulated radiation therapy (IMRT) is advantageous over 3-dimensional conformal radiotherapy in terms of avoiding adjacent organs-at-risk. A prospective phase I study found that IMRT was associated with a global decrease in normal tissue exposure compared to 3-dimensional conformal radiotherapy, and that the former as associated with a significant reduction in V20 (21.5% vs. 26.5%, *p* < 0.01) and MLD (11.9 Gy vs. 14.9 Gy, p < 0.01) compared to the latter [15]. Chun et al. performed a secondary analysis of the RTOG 0617 trial and found that patients in the IMRT group had significantly lower occurrences of grade ≥ 3 RP than did those in the 3-dimensional conformal radiotherapy group (7.9% vs. 3.5%, *p* = 0.039) [16].

In the near future, it might also be possible to use proton beam therapy to deliver a sufficient radiation dose without increasing lung toxicity. Berman et al. compared dose distributions between IMRT and intensity modulated proton therapy in 10 patients with pathologic stage IIIA NSCLC [17]. The CT treatment planning scans for IMRT (50.4 Gy in 1.8 Gy fractions to the target volume) were used, and the average MLDs, when using IMRT and proton therapy, were 10.83 Gy and 6.675 Gy, respectively. Hence, they were able to reduce the MLD to below 10 Gy, which was the cut-off value in our study by using IMPT. Such newer technologies may enable MLD reductions to the lowest doses possible; however, it remains necessary to perform prospective studies to compare the effectiveness and lung toxicity profiles of IMRT and intensity modulated proton therapy for induction CCRT.

To the best of our knowledge, our study is the first to show that MLD and tumor location are significant predictors of RP in patients undergoing induction CCRT for NSCLC. However, our study had certain limitations. First, it was a retrospective analysis in which certain patient details were unknown; for example, Kocak et al. reported that RP scoring was difficult in 28% of their patients because of confounding medical conditions [18]. Second, although induction CCRT was administered to patients judged to be operable by the surgeon, detailed assessments on the underlying lung disease using CT were not made. For example, Tujino et al. found that the pulmonary fibrosis score as determined via baseline CT was a better predictor of grade ≥ 2 RP than V20 alone according to receiver operating characteristic analysis [19]. Third, limited information was available regarding internal target volume, surgical invasiveness, and surgical morbidity due to the retrospective nature of the study.

## Conclusions

MLD and lower lobe location were predictors of grade ≥ 2 RP in patients with NSCLC who received induction CCRT. Therefore, it is necessary to reduce MLD during radiotherapy planning to the greatest extent possible to prevent the occurrence of this adverse event.

## Data Availability

The data will not be shared because the ethics committees did not allow sharing of the data.

## Abbreviations

CCRT: Concurrent chemoradiotherapy
CRT: Chemoradiotherapy
CT: Computed tomography
CTCAE: Common Toxicity Criteria for Adverse Events
DVH: Dose-volume histogram
GTV: Gross tumor volume
IMRT: Intensity-modulated radiation therapy
MLD: Mean lung dose
NSCLC: Non-small cell lung cancer
OS: Overall survival
RP: Radiation pneumonitis
Vx: Percentage of the lung volume that received more than x Gy
